# The effect of teaching puberty health concepts on the basis of a health belief model for improving perceived body image of female adolescents: a quasi-experimental study

**DOI:** 10.1186/s12889-020-08482-2

**Published:** 2020-03-20

**Authors:** Maasoumeh Barkhordari-Sharifabad, Saeed Vaziri-Yazdi, Mansoureh Barkhordari-Sharifabad

**Affiliations:** 1 Department of Nursing, School of Medical Sciences, Yazd Branch, Islamic Azad University, Shohadaye Gomnam Blvd., Safaiyeh, Yazd, Iran; 2 Department of Educational Sciences, Yazd Branch, Islamic Azad University, Yazd, Iran

**Keywords:** Body image, Puberty health, Health belief model, Health education

## Abstract

**Background:**

As children approach adolescence, they focus increasingly on their appearance and physical attraction due to teenage body-image. Teaching the concepts of adolescent health changes an individual’s attitudes towards parts of the body. The health belief model (HBM) is one of the significant pedagogic models in health education. According to this model, the individual’s decision and motivation for adopting healthy behaviors depends on three separate categories “personal perception, adaptive behaviors, and probability of performing that action or behavior”. This study investigated the effect of teaching puberty health concepts on the basis of a HBM on perceived body image in female adolescents.

**Methods:**

A quasi-experimental research design was used in the study. This study was conducted with 60 sixth grade girls in state elementary schools in Yazd, Iran, that were selected with cluster sampling method and assigned randomly into experimental and control groups. The experimental group were educated in the school during eight 45-min sessions based on the HBM, whereas the control group were educated using the traditional lecturing method. The data were collected with demographic and self-body image questionnaires completed before and after intervention. The data were analyzed with SPSS16 using analysis of covariance (ANCOVA).

**Results:**

The mean age of the participants was 12.16 ± 0.74 years. The findings showed that “perceived body image” and “students’ self-attitude” improved significantly after intervention; yet, no significant difference was found between the subscales “attitudes towards weight” and “satisfaction with various parts of the body”.

**Conclusion:**

The results of the study confirmed the efficacy and efficiency of teaching puberty health on the basis of the HBM on improving perceived body image in female adolescents.

## Background

Body image is a multi-dimensional structure referring to how people think, feel and behave toward their physical characteristics. Therefore, the three dimensions of body image include evaluation, emotions, and investment (cognition). Evaluation dimension of the body image refers to the satisfaction or dissatisfaction of individuals with their bodies and their assessors’ beliefs about it. The emotional dimension of the body image pertains to the feeling of anxiety, distress and other emotions associated with the body, and the investment dimension of body image refers to the cognitive, behavioral, and affective importance of body for self-evaluation [1–3]. An individual’s perception of their body may change regarding the context in which it operates [4]. Body dissatisfaction refers to the negative thoughts and feelings about one’s body and the perceived difference between the current and the ideal body size [5]. The distortion of the body image refers to the incorrect perception of body shape or size [6]. Body image and an attractive appearance is more important among adolescents than other classes of the society [7].

On the other hand, sociocultural factors, especially perceived pressure from others and comparing oneself with others, influences body image [8]. Over the last decades, Iranian girls have been greatly concerned with body slimness and fitness. Satellite propaganda and also commercial ads in health and beauty magazines contain a huge volume of ads on slimness and fitness [9].

Adolescence begins with puberty and is characterized by significant changes in hormonal levels and physical appearance (e.g. rapid physical growth, changes in the face structure and appearance of secondary sexual characteristics), and psychological and social characteristics [10, 11]. Adolescence is associated with a specific vulnerability to mental image disorder [7], and body dissatisfaction is prevalent among adolescents [1]. During these years, girls develop more problems in relation to body image than boys [1, 12], i.e., although maturity draws boys closer to the ideal body of men in terms of culture, it draws girls away from the current ideal fitness and raises concerns about body image [13]. Studies indicated that body satisfaction is low in 46% of girls and 6% of boys; also, 59% of overweight girls and 48% of overweight boys showed low satisfaction with physique [14]. Underweight girls have more body satisfaction than those with normal weight and those overweight, while being underweight and overweight in boys is associated with lower body satisfaction [15, 16]. According to research, body image disorder is associated with eating disorders, psychiatric distress, performance disorders, sexual dysfunction, depression, low self-esteem, preoccupation with appearance and cosmetic surgery [13, 17–21].

Furthermore, changes during puberty contribute to the critical period adolescence brings leading to probable anxiety [22]. Some studies in this field demonstrated that 61.7% of female students experienecd moderate to severe anxiety during their puberty [23]. Additionally, anxiety in the onset of adolescence may predispose teenagers to sensitivity in interpersonal relations and reduced compatibility [22]. Puberty health and awareness of the causes of special changes that occur during this period can reduce the incidences of many problems that can happen during this period and thereby diminish anxiety [23].

School-based educational programs were proposed to help prevent eating disorders and improve the body image of female adolescents. School environments are suitable places to implement health promotion programs, because adolescent students are available and have incentives to participate in educational activities [24]. One of the education models raised in health education is the HBM that reflects the willingness of individuals to perform health-promoting behaviors [25, 26]. According to this model, individuals must first feel threatened by the subject matter (perceived sensitivity). Then, they must perceive the depth of the risk and the severity of its various complications in their physical, social, psychological and economic dimensions (perceived severity). Then, with the positive signs that they receive from their environment (cues to action), they should believe in the usefulness and applicability of the educational programs (perceived benefits). They should also consider the deterrent factors of cues to action less costly than its perceived benefits (perceived barriers) and feel efficient and sufficient in overcoming barriers to behavior change (self-efficacy). So, after these steps, they can ultimately take preventive measures [27].

The effect of education based on this model was investigated in various studies. For example, a study conducted by Shirzadi et al. indicated a significant positive correlation between perceived sensitivity, perceived benefits, perceived barriers, cues to action, and increased puberty health in adolescent girls. Also, the perceived benefits, perceived barriers, and cues to action are predictors of physical puberty health behaviors [26]. The findings of the study by Soliman et al. (2018) in Palestine indicated that using the health belief model was effective in improving the knowledge and performance of people at high risk of obesity and making changes in health behavior [28]. The results of Salem et al. (2018) in Egypt also indicated that education based on the HBM not only enhanced the nutritional knowledge of female students, but also improved their eating habits [29]. The findings of another study indicated that the effectiveness of education intervention based on the HBM was effective in students, adopting preventive and controlling behaviors in the principles of puberty health [30].

Regarding the importance of the subject and the stated issues, the present study aimed at answering the question of whether education about puberty health concepts based on the HBM improves perceived body image of female adolescents.

## Methods

### Design and samples

This was a quasi-experimental pretest-posttest research with a control group that was performed in 2019. The statistical population comprised all sixth grade primary school female students in Yazd, Iran. The research setting was state elementary schools in Yazd, Iran, that were selected with cluster sampling method. Sixty subjects were selected by convenience sampling, and then randomly assigned into two groups of thirty subjects; control and experimental. The age range of participants was 11 to 13 years old and their mean age was 12.16 ± 0.74 years. Given that previous studies proposed the age of 12 as the best age for teaching puberty health [31] and considering that the mean age of the onset of menstrual cycle is reported to be 13 years old in Iran [31, 32], sixth grade primary school girls were selected as participants. The inclusion criteria were: having had at least three menstrual periods and inclination for participation in the study.

Students of both groups completed the Multidimensional Body–Self Relations Questionnaire (MBSRQ) as a pre-test and post-test. The experimental group was educated in the school during eight 45-min sessions based on the HBM, whereas in the control group, concepts of puberty health were taught using the conventional lecturing method.

### Procedure

On the basis of need, assessment and goals preset by the Iranian Ministry of Education and Training, eight 45-min educational sessions were developed by the researcher (the third author) to be completed during 1 month. According to the HBM, the first three sessions formed the awareness stage providing the required knowledge on puberty and its related hygienic issues. The next three sessions dealt with sensitivity and its perceived severity on the basis of the model. These sessions addressed the specific risks and consequences and prevalent diseases induced by lack of observing puberty health and also the depth of these risks and sensitivity of the complications. The seventh session formed the aspect of perceived benefits that showed advantages bestowed on the individual by adopting preventive behaviors. Finally, the eighth session formed the aspect of perceived barriers that reduce performance of preventive behaviors by the individual (Table 1).

**Table 1:** Summary of the education session structure based on the HBM

Steps of the HBM	Number of education sessions	The procedure of sessions
Awareness stage	3 sessions	Stages of maturity, how to wash and bathe, the type of nutrition at this time, complications of anemia, how to reduce complications when taking iron tablets, the effect of routine and light exercises, etc.
Perceived sensitivity stage	2 sessions	Complications and risks resulting from anemia, complications of not taking iron tablets, suffering with infectious and genital diseases and urinary tract infections, complications related to anemia due to non-consumption of nutritional and iron fortifying ingredients, excessive obesity and thinness, infectious rashes due to lack of care of the skin, etc.
Perceived severity stage	1 session	Special consequences and common illnesses due to the failure to observe puberty health such as iron deficiency, anemia, improper washing, not bathing and manipulating skin rashes, etc.
Perceived benefit stage	1 session	The positive benefits of observing hygiene during puberty such as not suffering from anemia due to iron tablet supplementation and nutritious foods, lack of suffering from severe genital and urinary infections due to proper washing and bathing, relieving severe pain in the abdomen and lower back due to normal and light sports activities, the lack of complications due to obesity and excessive weight loss in the event of observing a proper diet and gradual improvement of facial rashes if not rubbed, etc.
Stage of perceived barriers	1 session	Barriers such as economic problems to produce iron tablets etc.

It should be noted that according to the National Iranian Survey, the prevalence of iron deficiency anemia is 39% among adolescent girls. Accordingly, the Iranian Ministry of Health recommends Ferro sulfate 1 tab/week for a 4-month period per year. Since the onset of the educational year is the most suitable time of tablet administration, thus, education in this field was considered as part of puberty health education for adolescents (http://ssu.ac.ir/cms/index.php?id=2299).

### Measures

The data collection tool in this study consisted of two parts.

1. Demographic characteristics questionnaire including age, mother’s academic degree, father’s academic degree, mother’s job and father’s job.

2. Multidimensional Body–Self Relations Questionnaire (MBSRQ): This questionnaire is used to evaluate the person’s body image and is a 68-item scale developed by Cash et al. in the early 1980’s [33, 34]. The total score of this inventory, which uses a 5-point Likert scale, ranges between 68 and 339. This tool includes 3 subscales: “self-attitude”, “attitude towards weight” and “satisfaction with various parts of the body”.

In the self-attitude subscale, there are three prominent physical aspects, namely, physical appearance, physical fitness, and health, each of which includes two areas of evaluation and understanding (evaluation and perceiving appearance, evaluation and perception of physical fitness, evaluation and perception of health). Individuals showed the level of their agreement with each item in terms of a 5-point Likert scale ranging from 1 indicating definitely disagree to 5 indicating definitely agree. The scores in the subscale ranged between 54 and 270. A higher score was indicative of higher satisfaction.

In the subscale of “satisfaction with various parts of the body” such as face, upper trunk, trunk and lower trunk, muscular consistency, weight, height, and general appearance, 1 indicated completely dissatisfied and 5 indicated completely satisfied. The scores in the subscale ranged between 8 and 40.

The subscale of “attitude towards weight” includes “mental obsession and individual’s self-assessment of weight”. Mental obsession shows anxiety with obesity and attention to body weight, nutritional diet, and eating controlled behavior. The scores in the subscale ranged between 6 and 29.

The validity of the main sections of the questionnaire was reviewed and verified by Brown, Cash and Mikulka (1990), and showed a reliability coefficient of 81% [35]. The Multidimensional Body–Self Relations Questionnaire is a standard questionnaire used in various studies [36–39]. According to previous studies, this questionnaire is acceptable in Iranian samples according to psychological fitness [40–44]. Rahati (2007) investigated the validity of the multi-dimensional self-image questionnaire on Iranian adolescents. The results suggested acceptable validity of the tool. Also, Cronbach’s alpha coefficient of the questionnaire was 0.85 for female participants [41], indicating that the instrument had an acceptable confidence level.

### Data analysis

Data analysis was conducted using SPSS16. Statistical analysis was conducted using descriptive statistics (frequency and percentage, mean and standard deviation) and inferential statistics (univariate covariance).

## Results

In the present study, a total of 60 people participated in the study, and there was no subject attrition. The mean age of the participants was 12.16 ± 0.74. The youngest of participants was 11. For the majority of the participants, 71.7% of fathers and 76.7% of mothers held a diploma or a higher degree, 78.3% of mothers were homemakers and 46.7% of fathers were self-employed. The two groups were identical in terms of demographic characteristics.

Table 2 indicates the mean and standard deviation of body image scores and their components in the experimental and control groups separately in the pre-test and post-test phases (Table 2).

**Table 2:** Descriptive indices of body image and its components in the two experimental and control groups

Variable	Experimental group N = 30		Control group N = 30	
	Mean	SD	Mean	SD
**Body image**				
Pretest	244.66	25.67	229.93	25.06
Posttest	273.06	26.83	251.40	28.93
**Satisfaction with various parts of the body**				
Pretest	32.26	5.42	30.26	6.37
Posttest	34.10	5.22	31.80	5.89
**Attitude towards weight**				
Pretest	17.90	2.65	17.20	3.48
Posttest	18.23	2.62	17.50	2.52
**Self-attitude**				
Pretest	186.10	21.99	186.00	26.21
Posttest	206.46	24.30	201.33	25.57

Significant differences were found between groups in pretest scores. Therefore, pretest scores became covariates in subsequent analysis and the research questions were answered using ANCOVA. To use covariance, some assumptions were considered. Therefore normality of data distribution was assessed using Kolmogorov-Smirnov test (*P* > 0.05). Then, Levene’s test was used to establish equality of variances and the result confirmed this. Also, the homogeneity of regression slope was established.

In order to study the effect of educational intervention using HBM, the ANCOVA was used. The results are displayed in Table 3.

**Table 3:** The summary of the results of ANCOVA on the effect of education of the concepts of puberty health based on HBM on body image and its components

Variable	Sum of Squares	df	Mean Square	F	Sig.	Eta Squared	Observed Power
**Body image**	3610.088	1	3610.088	4.617	0.036	0.075	0.561
**Satisfaction with various parts of the body**	3.155	1	3.155	2.003	0.162	0.034	0.285
**Attitude towards weight**	6.199	1	6.199	0.939	0.337	0.016	0.159
**Self-attitude**	5029.843	1	5029.843	8.528	0.005	0.130	0.819

As a result of the ANCOVA in Table 3, a significant difference was found between the groups on “body image” (F = 4.617, *P* = 0.036) and “self-attitude” (F = 8.528, *P* = 0.005). Therefore, the education of the concepts of puberty health based on the HBM was effective in improving “body image” and “self-attitude”.

Considering the results of the table above, no significant difference was found between groups on the subscales of “satisfaction with various parts of the body” (F = 2.003, *P* = 0.162) and “attitude towards weight” (F = 0.939, *P* = 0.337) (Table 3). Therefore, it can be concluded that the intervention was not effective on improving “satisfaction with various parts of the body” and “the students’ attitude towards their weight”.

## Discussion

The present study aimed at investigating the effectiveness of educating the concepts of puberty health based on HBM for improving body image in female students. Based on the results of this study, the scores of the satisfaction dimension increased after the educational intervention in both groups, but there was no significant difference between the two groups after intervention. The study by Hinz indicated that provision of an educational program to help boys and girls to better accommodate themselves to inevitable changes in puberty reduces the risk of dissatisfaction among the students [45]. Futhermore, the findings of the present study were not consistent with the results of Abedi-Parija et al. (2017), where part of their intervention replaced negative beliefs with real and logical thoughts and cognitive restructuring and improving satisfaction with the body [46]. In a study by Cousineau et al. (2010), the body dissatisfaction prevention program and puberty health education were effective in reducing body dissatisfaction [47]. The reason for such inconsistency may be due to differences in the implementation of educational programs, the population and research tools.

One of the most important causes of body dissatisfaction in adolescent girls is an unrealistic ideal symbol of feministic beauty in recent years, which largely emphasizes weight loss and an idealized body. Since achieving the ideal body determined for most girls is inaccessible, it can lead to body dissatisfaction and consequently, to a variety of diseases, and physical, mental and social harms. This should be taken into consideration by policymakers, managers, teachers, and families to improve dissatisfaction through providing appropriate programs including appropriate educational programs.

After education, the attitude score of individuals increased concerning their weight in both groups, but there was no significant difference based on ANCOVA. In the study of McArthur et al. (2018), the HBM had low predictive power in predicting the body mass index [25]. Crockett also states that since puberty is a physiological process, adolescents ought to be taught to better perceive puberty changes and acquire a positive attitude towards body image. Behavioral change approaches should be used to promote selection of accurate choices with regard to various issues, especially body weight [48]. It is important to note that the overall appearance of individuals at this age is of particular importance to them, and proper education about increasing and decreasing body mass during this period can reduce this concern with emphasis on body swelling at certain times, e.g., menstruation.

The results of the current study indicated that the girls who received education on the concepts of puberty health based on the model had lower levels of concern about health, appearance and fitness than the girls who received routine education. These findings were consistent with the results of other studies [25]. The results of Salem et al. (2017) indicated that there was a significant difference in pre-test and post-test education based on HBM in improving health and body appearance [29]. *O’Neill* et al. (2016) investigated a curriculum based on principles of HBM and social learning theory in improving the fitness and safety of elementary school students. The results indicated that this curriculum was effective in promoting fitness and safety of students [49]. In explaining this finding, it can be pointed out that providing girls with puberty education based on the model, provides them with a clearer understanding of their bodies, enhances their self-esteem and self-confidence, and adds to their passion for life and motivation for success.

Based on the results of this study, there was a significant difference between the body image of the control and the experimental groups after intervention based on the HBM. The findings of this study were consistent with the results of other studies [46, 47, 50]. By focusing on perceptions, thoughts, and increasing beliefs about the usefulness and applicability of the program and understanding the obstacles, being educated on the puberty health concepts based on the HBM helps students to have a more realistic image of their bodies.

The self-reporting tool was a limitation of the present study, and similar to other self-reporting tools, there was the possibility of fatigue, and lack of sufficient opportunity. Thus, some contributors may have refused to provide real answers. The lack of control of intervening variables such as the different socio-cultural level of families was another limitation of this study. Hence, it is recommended that a similar study with controlled intervening variables be designed.

## Conclusion

Based on the results, teaching health concepts on the basis of HBM would improve perceived body image and self-attitude among female adolescents. Consequently, offering educational programs during puberty, especially for adolescent girls while raising their awareness, lays the groundwork for posing questions and solving the problems of puberty that can affect body image. Model-based education will have a greater effect on body image improvement by focusing on perceptions and enhancing beliefs about the applicability of the program and understanding the benefits and barriers.

## Data Availability

Sharing the data is not possible due to an agreement with the participants on the confidentiality of the data.

## Abbreviations

HBM: Health Belief Model
MBSRQ: Multidimensional Body–Self Relations Questionnaire,
ANCOVA: Analysis of covariance
